# Postprandial increase in serum CA125 as a surrogate biomarker for early diagnosis of ovarian cancer

**DOI:** 10.1186/s12967-018-1489-4

**Published:** 2018-05-01

**Authors:** Zhuowei Gu, Yifeng He, Yue Zhang, Mo Chen, Keqi Song, Yuting Huang, Qing Li, Wen Di

**Affiliations:** 1 Department of Obstetrics and Gynecology, Ren Ji Hospital, School of Medicine, Shanghai Jiao Tong University, 160 Pujian Road, Shanghai, 200127 China; 2 Shanghai Key Laboratory of Gynecologic Oncology, Ren Ji Hospital, School of Medicine, Shanghai Jiao Tong University, Shanghai, 200127 China; 3 Tumor Microenvironment and Metastasis Program, The Wistar Institute, University of Pennsylvania, Philadelphia, PA 19104 USA; 4 Department of Bioinformatics and Biostatistics, School of Life Sciences and Biotechnology, Shanghai Jiao Tong University, Shanghai, 200240 China; 5 Department of Gynecology, Obstetrics and Gynecology Hospital, Fudan University, Shanghai, 200011 China; 6 Children’s Research Institute, Children’s National Medical Center, Washington, DC 20010 USA; 7 State Key Laboratory of Oncogene and Related Genes, Shanghai Cancer Institute, Ren Ji Hospital, School of Medicine, Shanghai Jiao Tong University, Shanghai, 200127 China

**Keywords:** Ovarian cancer, CA125, Fasting, Postprandial, Increment, Glucose, Insulin, PI3K, Akt, Mesothelin

## Abstract

**Background:**

CA125 is a prevalently used serum biomarker for detecting ovarian cancer over the last three decades. However, it has a significant deficiency in screening for early-stage cancer. With the purpose of exploring an effective approach to improve its performance in early diagnosis, we investigated the postprandial fluctuation pattern of cancer-derived CA125 and the underlying mechanism.

**Methods:**

In two medical centers, 551 patients sonographically diagnosed with ovarian (adnexal) cysts (< 5 cm in diameter) were enrolled and divided into five disease groups (pelvic inflammatory cysts, retention cysts, endometrioma, benign/borderline cystadenoma and malignant cysts). The subtle differences in 1-h postprandial serum CA125 increases were compared between disease groups. A support vector machine (SVM)-based algorithm was used for refining the performance of CA125 postprandial increment. Ovarian cancer xenograft animal and cancer cell models were used to recapitulate the clinical findings and reveal the molecular basis of postprandial blood glucose and insulin in invoking the synthesis/secretion/re-absorption of CA125.

**Results:**

Patients with ovarian cancer presented the highest postprandial increment 13.3 ± 0.7% (mean ± standard deviation) among the five disease groups. Using a CA125 increment ≥ 10% criterion, the sensitivity, specificity, positive predictive value (PPV) and negative predictive value (NPV) reached 83.3, 96.3, 61.1 and 98.8%, respectively, for early-stage ovarian cancer. This performance was further improved by the SVM-based CA125-increment algorithm, which exhibited 91.7% sensitivity, 99.2% specificity, 89.2% PPV and 99.4% NPV. Both modalities manifested diagnostic advantages over the traditional CA125 test (75.0% sensitivity, 25.4% specificity, 6.6% PPV and 93.6% NPV at the cut-off of 35 U/mL). Regarding the molecular basis, the postprandial blood glucose and insulin-invoked overexpression of Mucin 16 (encoding CA125) were demonstrated in animal and cancer cell models, which were mediated by the PI3K-Akt pathway. Nevertheless, a Mesothelin-based CA125 re-absorption behavior was noted in the treated cancer cells, which contributed to the over-drop following the postprandial peak of serum CA125.

**Conclusions:**

Cancer-derived serum CA125 possesses a unique and distinctive postprandial pattern, that distinguishes it from the common CA125 elevation in a benign disease condition. The dynamic measurement/assessment strategy can achieve a discriminatory power superior to that of a static test.

**Electronic supplementary material:**

The online version of this article (10.1186/s12967-018-1489-4) contains supplementary material, which is available to authorized users.

## Background

Earlier diagnosis of ovarian cancer could significantly increase survival. The general 5-year survival rate of ovarian cancer patients is approximately 90% at stage I but only 5–60% at stage II or above [1]. Located within a deep pelvic cavity, this epithelial malignancy usually arises with no typical bodily signs or symptoms. Therefore, the current modalities for detecting early-stage ovarian cancer rely heavily on transvaginal ultrasonography (TVU) and serum biomarkers, such as CA125 [2]. Unfortunately, a malignant ovarian cyst with a diameter < 5 cm is difficult to diagnose via TVU and is usually morphologically indistinguishable from a benign cyst, e.g., serous/mucinous cystadenoma, endometriotic and simple cysts of the ovary [3, 4]. Additionally, the cancer biomarker CA125 has limitations for discriminating early-stage ovarian cancer from benign diseases [4, 5]. For example, it is difficult to determine whether elevated serum CA125 within the range of 35–65 U/mL is due to an unidentified ovarian cancer or a common CA125-secreting gynecological disease, such as a pelvic inflammatory cyst, endometrioma or cystadenoma [5]. Hence, there is a risk of missing the substantial progression of existing cancer and, subsequently, a poor prognosis. Notably, both ovarian endometriotic cysts and cystadenoma have odds of developing into endometrioid cancer (or clear cell cancer; incidence rate: 0.2–0.8%) [6] or cystadenocarcinoma (primarily serous carcinoma; incidence rate: 0.4–0.6%) [7], which complicates the clinical settings for early diagnosis and places more obstacles on the TVU and CA125-based screening programs.

Efforts to improve the efficacy of TVU and CA125-based screening include the adoption of more effective subsidiary/surrogate cancer biomarkers [8]. Of them, human epididymis protein 4 (HE4), an ectopically expressed sperm maturation-related protein, was found to be a potent substitute for traditional CA125 and is currently gaining popularity in gynecological oncology clinics [9]. Pre-clinical studies have indicated that serum HE4 exhibited higher sensitivity and specificity for detecting ovarian cancer than CA125 [10]. However, as more large-scale clinical trials were undertaken, the complexity of ovarian cancer and the variety of patient groups and reagent types need be considered in evaluating the efficacy of a serum HE4 test [11]. In a Quality Assessment of Diagnostic Accuracy Studies-2 (QUADA-2)-based meta-analysis by Li et al. it was revealed that HE4 just outperformed CA125 in diagnostic specificity (93% vs. 78%) while the sensitivities of both were similar (79% vs. 79%) [12]. More importantly, the area under the receiver operating characteristic (ROC) curve (AUC) was greater for serum CA125 than for serum HE4, which indicates that CA125 may have a superior diagnostic capacity if the cut-off is properly adjusted [12]. Additionally, Jacob et al. asserted that the clinical benefit of a combined serum HE4 and CA125 test is not significant if the additional cost is accounted for [13]. Therefore, the primary task for gynecological oncology researchers remains unchanged: to continue to explore more low cost, convenient and efficient subsidiary/surrogate biomarkers for early-stage ovarian cancer.

Both the synthesis and secretion rates of CA125 in ovarian cancer cells are keenly influenced by extracellular signals from circulating cytokines. Konishi et al. reported that the secretory levels of CA125 in cells cultured in vitro can be increased upon administration of epithelial growth factor (EGF) [14]. We, therefore, hypothesized that the CA125 glycoproteins produced in benign and malignant conditions might be distinguishable from one another according to their responses to stimulatory cytokines. In the human body, the insulin release pulse, which is induced by elevated postprandial blood glucose levels, comprises a natural extracellular cytokine signal source. Previous works have clarified that the survival, division and proliferation abilities of ovarian cancer cells are all affected by circulating insulin levels [15–17]. However, little is known about whether the postprandial pulse of insulin can induce a unique instant/transient waveform of serum CA125, by which the arsenal of diagnostic tools for clinicians to discriminate between benign and malignant ovarian cysts can be upgraded. Therefore, the aim of this study was to profile the characteristic patterns of postprandial fluctuations of CA125 in the peripheral blood associated with endometriosis and benign and malignant epithelial tumors of the ovary and to assess the applicability of utilizing postprandial increases in serum CA125 to detect early-stage ovarian cancer in a complex pathological condition.

## Methods

### Study population

From January 2015 through June 2017, patients were enrolled at two tertiary medical centers in Shanghai, China: The Department of Gynecological Oncology, Ren Ji Hospital, School of Medicine, Shanghai Jiao Tong University and The Department of Gynecology, Obstetrics and Gynecology Hospital, Fudan University. The inclusion criteria were (1) an ovarian (adnexal) tumor (cyst) ≤ 5 cm in diameter under ultrasonography; (2) a scheduled exploratory laparotomy. The exclusion criteria were (1) any elevated serum AFP, CA199, or CEA level; (2) an ultrasonography-confirmed dermoid cyst (teratoma) or other germ cell tumor; (3) computed tomography- or MRI-confirmed abdominal metastasis or metastatic gastrointestinal cancer; (4) late-stage or nonepithelial ovarian cancer demonstrated by postoperative pathology; (5) a history of diabetes mellitus.

### Cell culturing

The ovarian cancer cell lines SKOV-3, HO8910, ES-2, and OVCAR-3 were purchased from the Cell Bank, Chinese Academy of Sciences (Shanghai, China). Cells were cultured in DMEM (glucose = 1 g/L, Sigma-Aldrich, St. Louis, MO, USA) supplemented with 10% FBS (Thermo Fisher, Waltham, MA, USA) in 5% CO_2_ at 37 °C. The final concentrations of insulin (SinoBio, Shanghai, China), LY294002 (Selleck, Houston, TX, USA), AktVIII (Selleck), AZD6244 (Selleck), and CA125 standard (Thermo Fisher) were 100 nM, 20, 1 mM, 5 μM and 200 U/mL, respectively. The high glucose condition referred to a glucose concentration of 2 g/L.

### Xenograft animal model

To build the cancer xenograft model, ovarian cancer cells (1 × 10^6^) were subcutaneously injected into specific pathogen-free (SPF)-grade Balb/c nude mice weighting between 20 and 22 g. As tumors grew to 1 cm in diameter, mice were divided into four groups. (1) In the control group, each mouse was administered a dose of 0.2 mL saline p.o., and 0.2 mL saline i.h.; (2) in the glucose group, each mouse was administered 0.2 mL of 10% glucose p.o. and 0.2 mL saline i.h.; (3) in the insulin group, each mouse was administered 0.2 mL saline p.o. and 0.2 mL insulin (5 mU) i.h.; (4) in the glucose plus insulin group, each mouse was administered 0.2 mL of 10% glucose p.o. and 0.2 mL insulin (5 mU) i.h. The type I diabetes mouse model was built by administration of alloxan (Sigma-Aldrich) [18]. Specifically, for each tumor-bearing nude mouse, 120 mg/kg alloxan was first administered via intraperitoneal injection, followed by a second dose of 80 mg/kg alloxan after a 24-h interval. The blood samples of treated mice were collected on day 3 via the tail-cutting method after a 12-h fasting period. Plasma glucose ≥ 11.1 mmol/L indicated that the diabetes nude mouse model was successfully generated.

### CA125 assay

CA125 concentrations were detected in serum samples (fasting, postprandial or post-75 g oral glucose) from the patients (or animals) and in the supernatant (or cellular content) from cell cultures using the same reaction plate from a CA125 ELISA Kit (Thermo Fisher) according to the manufacturer’s instruction.

### Protein labeling and deglycosylation

The standard CA125 protein (1000 U/mL, Thermo Fisher) was labeled with Alexa Fluor 488 NHS Ester (Thermo Fisher) according to the manufacturer’s instruction. The labeled CA125 was visualized at 517 nm using a 488-nm excitation wave. Removal of N-linked oligosaccharides of CA125 protein was achieved by digesting the protein with PNGaseF (New England Biolabs, Ipswitch, MA, USA) for 1 h at 37 °C.

### RNA interfering

Mesothelin (MSLN)-specific and negative control (NC) siRNAs were purchased from GenePharma (Shanghai, China). MSLN-siRNA-1, forward: 5′-ccauuggaccugcugcuautt-3′, reverse: 5′-auagcagcagguccaauggtt-3′; MSLN-siRNA-2, forward: 5′-gccucaucuucuacaagaatt-3′, reverse: 5′-uucuuguagaagaugaggctt-3′. NC-siRNA, forward: 5′-uucuccgaacgugucacgutt-3′, reverse: 5′-acgugacacguucggagaatt-3′. siRNAs were transfected with Lipofectamine RNAiMAX (Thermo Fisher) according to the manufacturer’s instruction.

### Reverse transcription (RT)-quantitative polymerase chain reaction (qPCR)

Total RNA was isolated from the cultured cells using TRIzol (Thermo Fisher) and then reverse-transcribed into cDNAs using a Universal cDNA Synthesis Kit (Exiqon, Vedbaek, Denmark). Real-time PCR was performed using the primer pairs for Mucin 16 (forward: 5′-ccccaaattccagaggtgaa-3′, reverse: 5′-tgacaaaggcgcactggtac-3′), MSLN (forward: 5′-cgccttgctttccagaacat-3′, reverse: 5′-attctgctgactgagcgcct-3′) and β-actin (ACTB, forward: 5′-agcgagcatcccccaaagtt-3′, reverse: 5′-gggcacgaaggctcatcatt-3′). SYBRGreen I dye (Thermo Fisher) served as a quantitative indicator in the qPCR reaction. An ABI PRISM 7900 Sequence Detection System [Applied Biosystems, Carlsbad, CA, USA) was used for PCR experiments. The cycle threshold (CT) of each qPCR assay was recorded, and the results were presented as 2^−ΔCT^ (ΔCT = CT(target) − CT(ACTB)].

### Western blotting

The primary antibodies used for recognizing the target proteins were as follows: mouse anti-human Mucin 16 monoclonal antibody (1:10, Cat. No. sc-365002, Santa Cruz, Dallas, TX, USA), mouse anti-human Mesothelin monoclonal antibody (1:10, Cat. No. sc-365324, Santa Cruz) and mouse anti-human β-actin monoclonal antibody (1:10, CW Biotechnology, Beijing, China), which were then detected using the secondary antibody provided by the Wes Mouse Master kit (ProteinSimple, San Jose, CA, USA). All the Western blotting experiments were performed using Wes (ProteinSimple). The intensities of the resultant bands were quantified and visualized using the “Compass for SW” software (ProteinSimple).

### Statistics

Two-sided χ^2^ test/Fisher’s exact test and Student’s t test (paired-samples or independent-samples; and two-sided independent-samples t test was the default setting) were used for nominal and numerical data, respectively. A support vector machine (SVM) was generated using the “kernlap” package in R software (version 3.3.3). The sensitivity of a diagnostic criterion was defined as the percentage of correctly identified cases among all pathologically confirmed ovarian cancer cases. The specificity was defined as the percentage of correctly identified cases among all pathologically confirmed non-cancer cases. The positive predictive value (PPV) was defined as the percentage of correctly identified cases among all the cases that could be judged as ovarian cancer by a given diagnostic criterion. The negative predictive value (NPV) was defined as the percentage of correctly identified cases among all the cases that could be judged as benign diseases by a given diagnostic criterion. SPSS 18.0 software (IBM, Armonk, NY, USA) was used to complete the statistical analyses (including the ROC analysis). p ≤ 0.05 was considered significant. All animal and in vitro experiments were carried out in triplicate unless otherwise indicated.

## Results

### The postprandial serum CA125 is significantly elevated in ovarian cancer patients

**Fig. 1:**
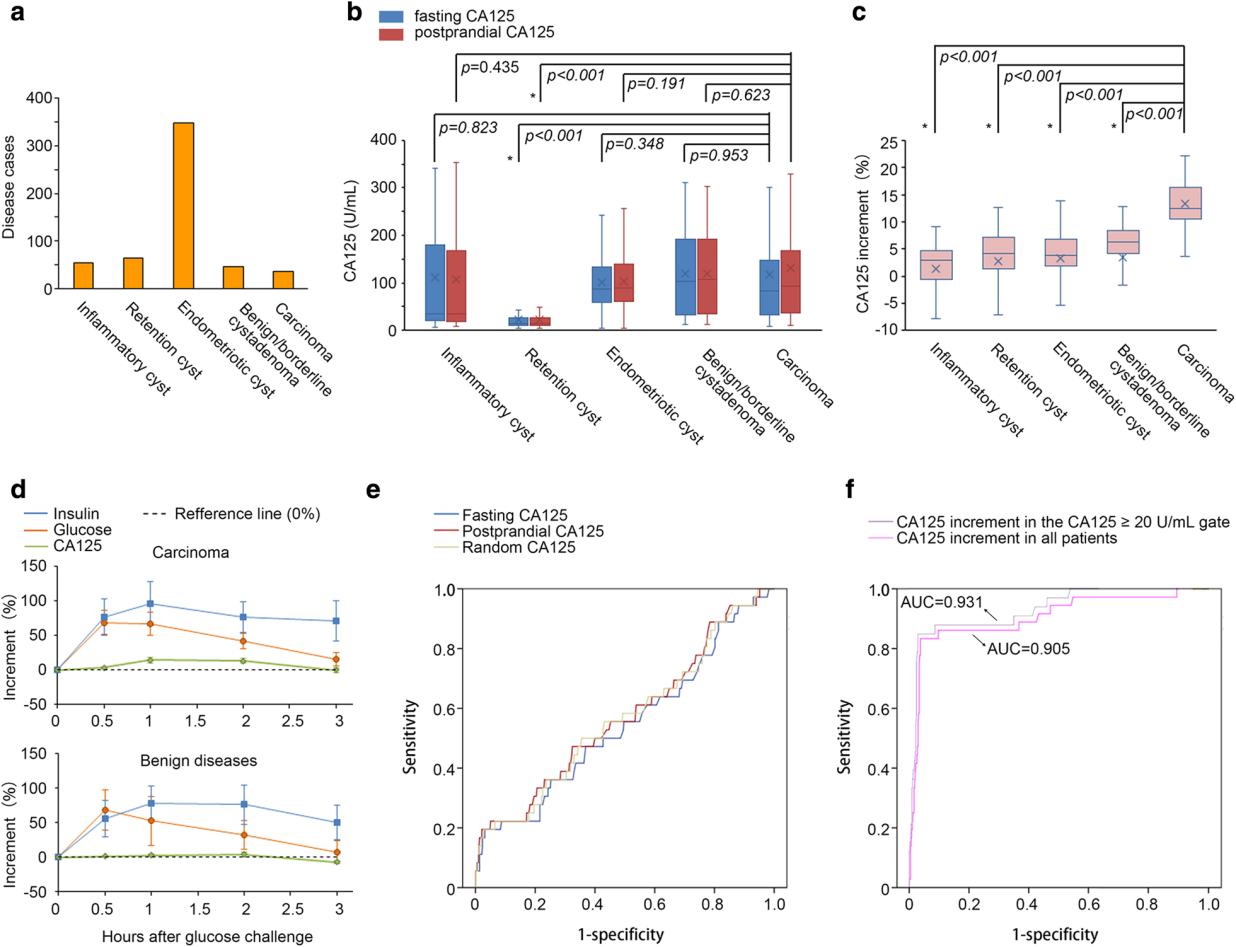
The clinical, metabolic and diagnostic characteristics of increases in postprandial serum CA125. **a** Disease distribution characteristics of the enrolled patients. **b** Boxplots for the fasting and postprandial CA125 levels of the five disease groups. **c** Boxplots for the postprandial CA125 increases of the five disease groups. **d** Metabolic characteristics of blood glucose, serum insulin and CA125 in the cancer and non-cancer patients. **e** ROC curves of random, fasting and postprandial CA125 in the study population. **f** ROC curves of the CA125 increment in the CA125 ≥ 20 U/mL gate and in all patients of the study population (i.e., CA125 ≥ 0 U/mL gate). *Statistical significance (two-sided Student’s t test)

A total of 551 patients sonographically diagnosed with cysts (tumors) of the ovary (or adnexa) were enrolled (Additional file 1: Table S1). The average age of the patients was 40.1 ± 10.4 (mean ± standard deviation) years, and the average size of the tumors was 4.3 ± 0.5 mm in diameter. The patients’ serum CA125 levels (random) ranged from 5.9 to 1116.5 U/mL (97.3 ± 86.7 U/mL, Additional file 1: Table S2). The postoperative pathological diagnoses were 55 cases of sactosalpinx (pelvic inflammatory cyst), 65 cases of simple/mesonephric (retention) cyst, 348 cases of endometriotic cyst (ovarian endometrioma), 42 cases of serous cystadenoma, 3 cases of mucinous cystadenoma, 2 cases of borderline serous cystadenoma (Stage I), 29 cases of serous cystadenocarcinoma (Stages I–II), 1 case of mucinous cystadenocarcinoma (Stage I), 4 cases of endometrioid carcinoma (Stages I–II), and 2 cases of clear cell carcinoma (Stages I–II) (for disease distribution characteristics, see Fig. 1a). Clearly, ovarian endometrioma, with the highest number of cases, was the most important disease type to be determined as a benign cyst by TVU and/or CA125. However, except for patients with a retention cyst, serum CA125 levels of the other four disease groups (inflammatory cyst, endometriotic cyst, benign/borderline tumor and early-stage malignant cyst) were similar, which could not be discriminated from one another before the surgery (Additional file 1: Table S3).

To test whether the postprandial fluctuation in serum CA125 can differentiate a benign from a malignant source of the cyst, each patient underwent a fasting serum CA125 test and a 1-h postprandial serum CA125 test as they entered the ward. A portion of the patients was randomly selected (17 cancer patients; 33 non-cancer patients) for a 75-g oral glucose tolerance test (OGTT), among whom multipoint (0, 0.5, 1, 2 and 3 h) serum insulin and CA125 tests were additionally performed. We observed that 1-h postprandial CA125 levels were generally higher than fasting CA125 levels in the disease groups (for details, see Additional file 1: Table S4), except for in patients with an inflammatory cyst (Fig. 1b). Importantly, the ovarian cancer group exhibited the highest postprandial increment (increment = postprandial CA125 − fasting CA125) in CA125 level, which reached 13.3 ± 0.7% (p < 0.001 for the difference between fasting CA125 and postprandial CA125 in this group, paired-samples Student’s t test) and was significantly higher than the remaining four groups (p < 0.001 for all, independent-samples Student’s t tests, Fig. 1c). To investigate the detailed characteristics of the postprandial CA125 fluctuations, data from multipoint glucose, insulin and CA125 tests were collected. We noted that the ascending phase of the CA125 curve generally lagged behind that of the blood glucose curve, which sped up and reached its maximum as the insulin peak emerged; moreover, at the descending phase of the CA125 curve, a segment that dropped below the fasting level (over-drop) was observed in both groups (benign vs. malignant cysts, Fig. 1d and Additional file 2: Figure S1). Compared with that of benign cysts, the CA125 curve of malignant cysts presented an earlier peak with a higher increase in the ascending phase as well as a delayed, shortened and shallow over-drop segment in the descending phase (Fig. 1d).

We next evaluated the efficacies of random, fasting and postprandial CA125 tests in discriminating malignant cysts. Unfortunately, none of these tests yielded a satisfactory performance in early-stage ovarian cancer (Fig. 1e). The ROC curves of these tests were similar with AUCs of 0.568, 0.545 and 0.572, respectively, which approached the area corresponding to the null hypothesis of 0.5. Nevertheless, we noted that the sensitivity of CA125 could be elevated to > 90% if the cut-off was lowered to 20 U/mL (Additional file 1: Table S5). Therefore, we examined whether the postprandial increase in CA125 could serve as an additional index to improve the capacity to discriminate malignancies. The AUC corresponding to the CA125 postprandial increase was 0.931 in a CA125 ≥ 20 U/mL gate (Fig. 1f). The Youden’s index reached its maximum (0.819) when the increment cut-off was set to 10% (in the CA125 ≥ 20 U/mL gate). The sensitivity, specificity, PPV and NPV for this combined diagnostic criterion (i.e., CA125 ≥ 20 U/mL plus CA125 increment ≥ 10%) were 77.8, 97.5, 68.1 and 98.4%, respectively, which yielded a Youden’s index of 0.752 (for all enrolled patients, Table 1). This performance was better than that achieved by simply using CA125 ≥ 20 or ≥ 35 U/mL as the criterion (Table 1). Furthermore, we tested whether an increase in CA125 alone could be an index for early ovarian cancer detection. To our surprise, although the AUC corresponding to the postprandial increase was somewhat lower (0.905, Fig. 1f), the sensitivity, specificity, PPV, and NPV reached 83.3, 96.3, 61.1 and 98.8%, respectively, with a higher Youden’s index of 0.796 (for all enrolled patients) as the CA125 increment ≥ 10% criterion was adopted (Table 1). The clinical efficacy of the traditional CA125 test was thus improved, suggesting the possibility of using CA125 postprandial increment as a surrogate biomarker.

**Table 1:** Performances of static and dynamic serum CA125 test-based diagnostic criteria for early-stage ovarian cancer

Diagnostic criteria	Sensitivity (%)	Specificity (%)	PPV (%)	NPV (%)	Youden’s index
CA125 alone					
Random CA125 ≥ 35 U/mL	75.0	25.4	6.6	93.6	0.004
Fasting CA125 ≥ 35 U/mL	72.2	25.8	6.4	93.0	-0.020
Postprandial CA125 ≥ 35 U/mL	77.8	25.0	6.8	94.2	0.028
Random CA125 ≥ 20 U/mL	91.6	14.0	6.9	97.1	0.056
Fasting CA125 ≥ 20 U/mL	91.7	13.2	6.9	95.8	0.049
Postprandial CA125 ≥ 20 U/mL	94.4	13.0	7.1	96.0	0.075
CA125 postprandial increment alone					
CA125 increment ≥ 10%	83.3	96.3	61.1	98.8	0.796
CA125 and postprandial increment combined					
Fasting CA125 ≥ 20 U/mL and CA125 increment ≥ 10%	77.8	97.5	68.1	98.4	0.752
SVM-based CA125-increment algorithm					
σ = 25, fasting CA125 and postprandial increment as inputs	91.7	99.2	89.2	99.4	0.924

### An SVM-modified CA125 algorithm for detecting early-stage ovarian cancer

**Fig. 2:**
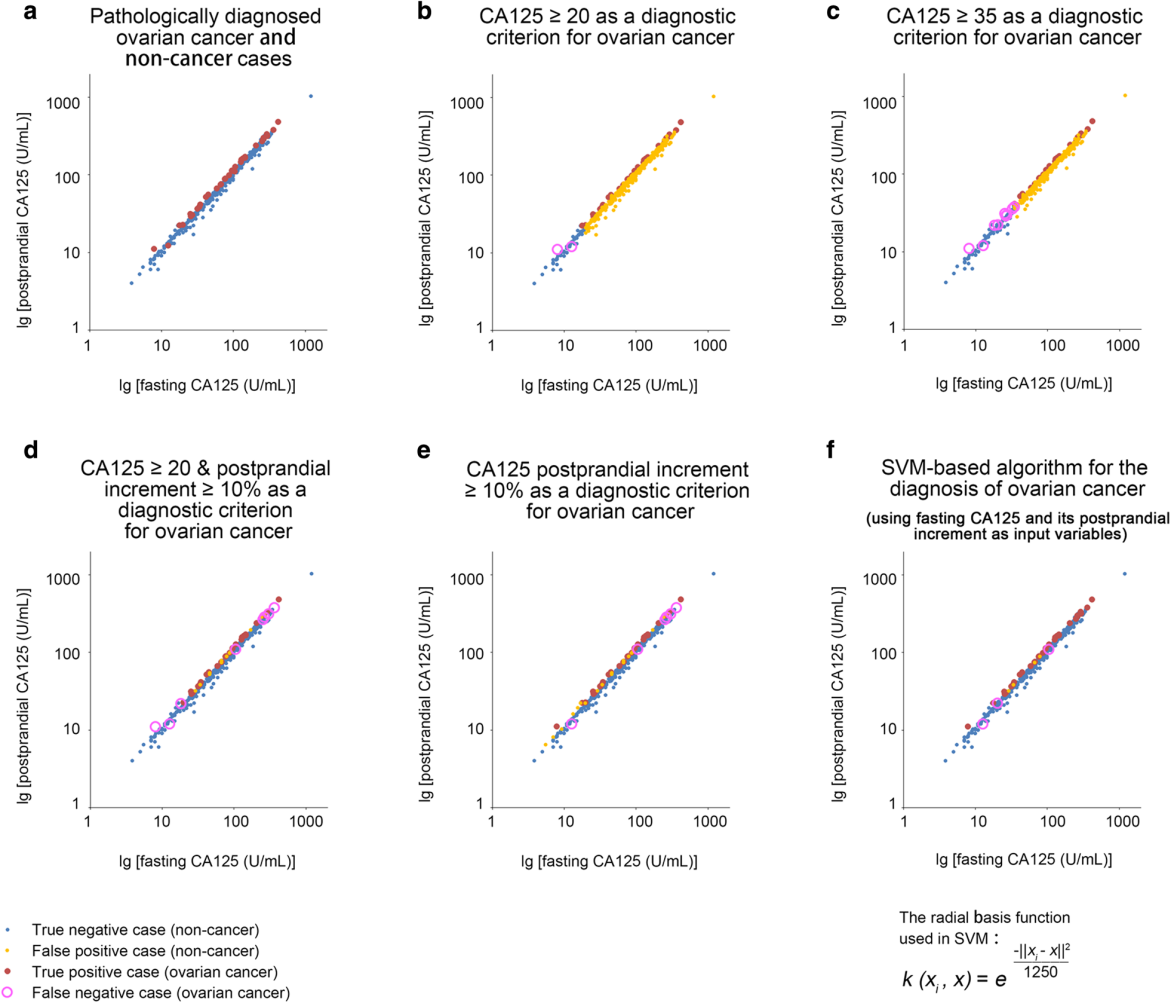
Performances of CA125-based diagnostic criteria in the study population. **a** The 2-dimensional distribution characteristics of fasting and postprandial CA125 in the enrolled patients. Most of the true positive cases (ovarian cancer cases, red dots) were located at the upper left margin of the scatter diagram, which exhibited higher postprandial increments in CA125 than the true negative cases (non-cancer cases, blue dots). **b** The diagnostic performance of the CA125 ≥ 20 U/mL criterion. **c** The diagnostic performance of the CA125 ≥ 35 U/mL criterion. **d** The diagnostic performance of the CA125 ≥ 20 U/mL plus CA125 increment ≥ 10% criteria. The number of false positive cases (orange dots) was greatly reduced by including an additional CA125 increment ≥ 10% criterion compared with the performance of the CA125 ≥ 20 U/mL criterion alone. **e** The diagnostic performance of the CA125 increment ≥ 10% criterion. The number of false negative cases (pink circles, 8 cases) was reduced (to 6 cases) by deleting the CA125 ≥ 20 U/mL criterion. **f** The diagnostic performance of the SVM-based CA125-increment algorithm (the exact form of the RBF kernel function is given). The false positive cases were reduced to 4 cases; and the false negative cases were reduced to 3 cases. The sensitivity (91.7%) and specificity (99.2%) for detecting early-stage ovarian cancer reached their maxima by using this algorithm

To examine whether the clinical efficacy of a CA125 postprandial increment test could be furthered refined, we constructed an SVM-based diagnostic algorithm. SVM can resolve non-linear classification and non-linear regression problems in biomarker discovery [19, 20]. We, accordingly, built a radial basis function (RBF)-based SVM model to investigate the possible non-linear variations in CA125 postprandial increments at different ranges of serum levels for early-stage ovarian cancer. The fasting CA125 and its postprandial increment were used as input parameters. Patient samples were divided into a training set and a validation set based on the leave-one-out method. The RBF parameter, σ, was confined to 25 with a penalty factor C of 100 following a cross-validation process using both training and validation sets (Fig. 2f). With the modified CA125 algorithm (Fig. 2f), we re-evaluated the diagnostic performance of CA125 postprandial increment in the early-stage ovarian cancer patients. The resultant sensitivity, specificity, PPV, and NPV were 91.7, 99.2, 89.2, and 99.4%, respectively, and an improved Youden’s index of 0.924 was achieved (Table 1), reflecting an advantage of the non-linear SVM method over the traditional linear method.

### Postprandial blood glucose and insulin levels are necessary for increased CA125

**Fig. 3:**
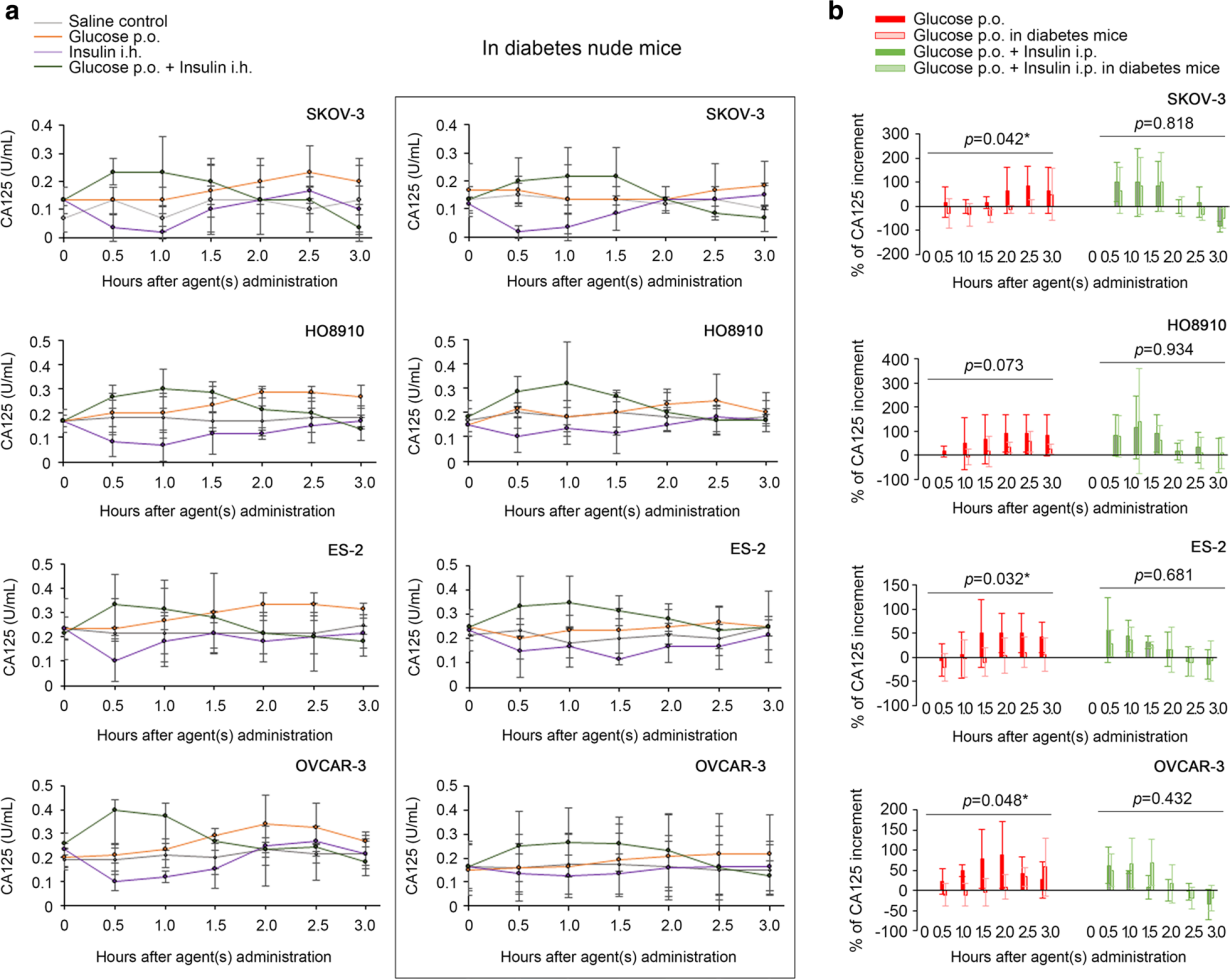
Postprandial serum CA125 fluctuations mimicked in animal models. **a** Shown are the time-dependent serum CA125 fluctuations after administration of saline, glucose, insulin or glucose plus insulin in SKOV-3, HO8910, ES-2 and OVCAR-3 xenograft nude mouse models. **b** Comparison of the percentages of serum CA125 increase after administration of glucose or glucose plus insulin among nude mouse models and diabetes nude mouse models. The oral glucose-induced increments in serum CA125 were significantly lower in the diabetes animal models than in the non-diabetes animal models. *Statistical significance (ANOVA)

As a translational research work, we expected to understand and demonstrate the respective roles of high glucose and insulin in the postprandial increase of serum CA125. Therefore, four ovarian cancer (SKOV-3, HO8910, ES-2 and OVCAR-3) xenograft animal models were built. In the xenografted nude mice, a slow ascending curve of the serum CA125 level was observed after administration of 20 mg oral glucose, and the peak of the curve emerged in approximately 2 h (peak time: 2.0 ± 0.4 h, Fig. 3a). Co-administration of glucose and insulin resulted in a faster peak in serum CA125, which emerged between 0.5 and 1 h (peak time: 0.6 ± 0.3 h), reflecting a synergistic effect of glucose and insulin on the increase in CA125 (Fig. 3a). When insulin was administered alone to the nude mice, contrary to the aforementioned increase in CA125, we observed a decrease in CA125 serum levels. The descending curve reached its minimum between 0.5 and 1 h (minimum time: 0.9 ± 0.5 h). Moreover, in the type I diabetes nude mice (induced by alloxan), the glucose-induced serum peak of CA125 was postponed to 2–3 h later (peak time: 2.6 ± 0.3 h), and the peak level was significantly lowered (Fig. 3b). However, when insulin was co-administered, the serum CA125 curve resumed its peak times and peak levels in all four cancer xenograft diabetes animal models (Fig. 3b). Therefore, both blood glucose and insulin levels are necessary for postprandial increases in serum CA125; nevertheless, insulin alone may have served as an accelerator for the plasmatic clearance/re-absorption of CA125, quite different from its co-role with glucose.

### CA125 synthesis/secretion is modulated by the PI3K/Akt pathway

**Fig. 4:**
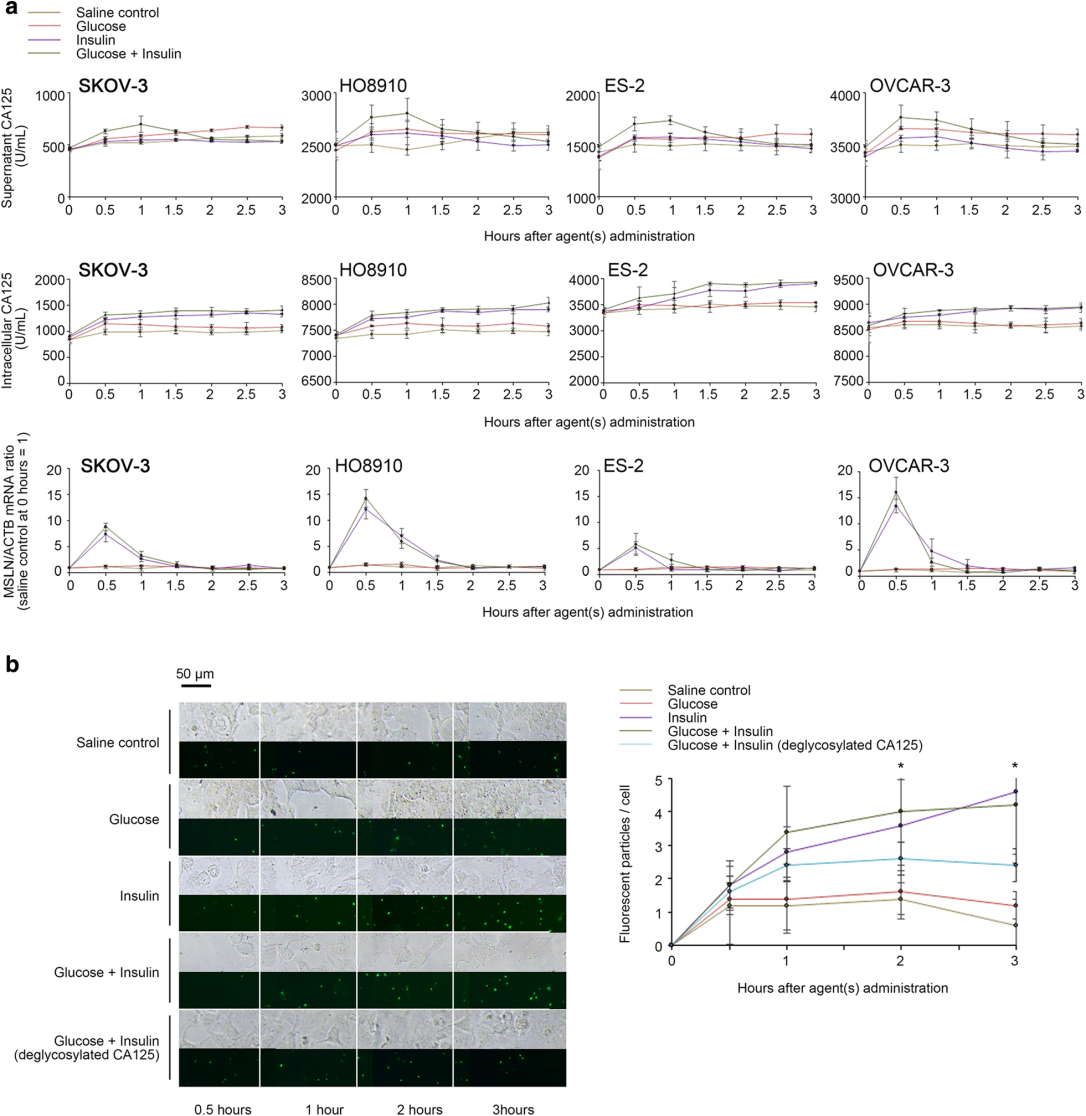
Glucose and insulin-invoked synthesis, secretion and re-absorption of CA125 in cancer cell models. **a** The time-dependent fluctuations of extracellular and intracellular CA125 levels and Mucin 16/ACTB mRNA ratios after administration of saline, glucose, insulin and glucose plus insulin in SKOV-3, HO8910, ES-2 and OVCAR-3 cancer cell lines, respectively (note: Mucin 16/ACTB mRNA ratios were set to 1 for saline controls). **b** The time-dependent re-absorption of Alexa Fluor 488-labeled CA125 in cultured ovarian cancer cells (SKOV-3) under saline, high-glucose, insulin-only, high-glucose plus insulin or CA125-deglycosylation conditions. The per cell fluorescent particles were presented as the mean of the particles counted in 20 randomly selected ovarian cancer cells under a ×200 microscopic field. The addition of insulin (i.e., the insulin-only and high-glucose plus insulin groups) significantly accelerate re-absorption of CA125 protein (green particles) into the cytoplasm of cultured cells, while the deglycosylation of CA125 protein partially blocked the re-absorption-promoting effect of insulin. *Statistical significance (ANOVA)

We re-validated the observed solo and synergistic effects of glucose and insulin on CA125 synthesis/secretion using in vitro cancer cell models (SKOV-3, HO8910, ES-2 and OVCAR-3). We noted that the addition of 2 g/L glucose to the culture medium only slightly increased CA125 concentration in the supernatant, while co-administration of glucose and insulin stimulated an active release of CA125 (Fig. 4a). An enhanced mRNA level of Mucin 16 (the coding gene of CA125) was detected in cells within 0.5 h after the co-administration of glucose and insulin, and the increase of supernatant CA125 level emerged at an adjacent time interval (0.5–1 h, Fig. 4a). Simply administrating insulin to the culture medium resulted in an inadequate increase (compared with the effects of co-administration of glucose and insulin), and later, a decrease in supernatant CA125 levels; however, intracellular CA125 and Mucin 16 mRNA levels steadily increased (Fig. 4a), suggesting divergent mechanisms for intracellular and extracellular CA125 metabolism. We postulated a dual role of insulin in the release and re-absorption of CA125. Therefore, CA125 protein was labeled with Alexa Fluor 488 to examine whether insulin could stimulate re-absorption of CA125 into cells. As expected, after administration of insulin, increasing fluorescent particles were found in the cytoplasmic area of the treated cells as time progressed, independent of the presence of glucose, which were significantly higher in number than those observed in the cells under a pure high glucose condition (Fig. 4b). It has been reported that, Mesothelin, a cell surface protein widely expressed in ovarian malignancies, can capture free CA125 with high affinity, where the N-linked oligosaccharides of CA125 are required [21, 22]. As PNGaseF-digested (deglycosylated) CA125 protein was added to the medium, we witnessed a decrease in the number of fluorescent particles endocytosed (Fig. 4b), implying the necessity of a specific binding to Mesothelin for the observed re-absorption.

**Fig. 5:**
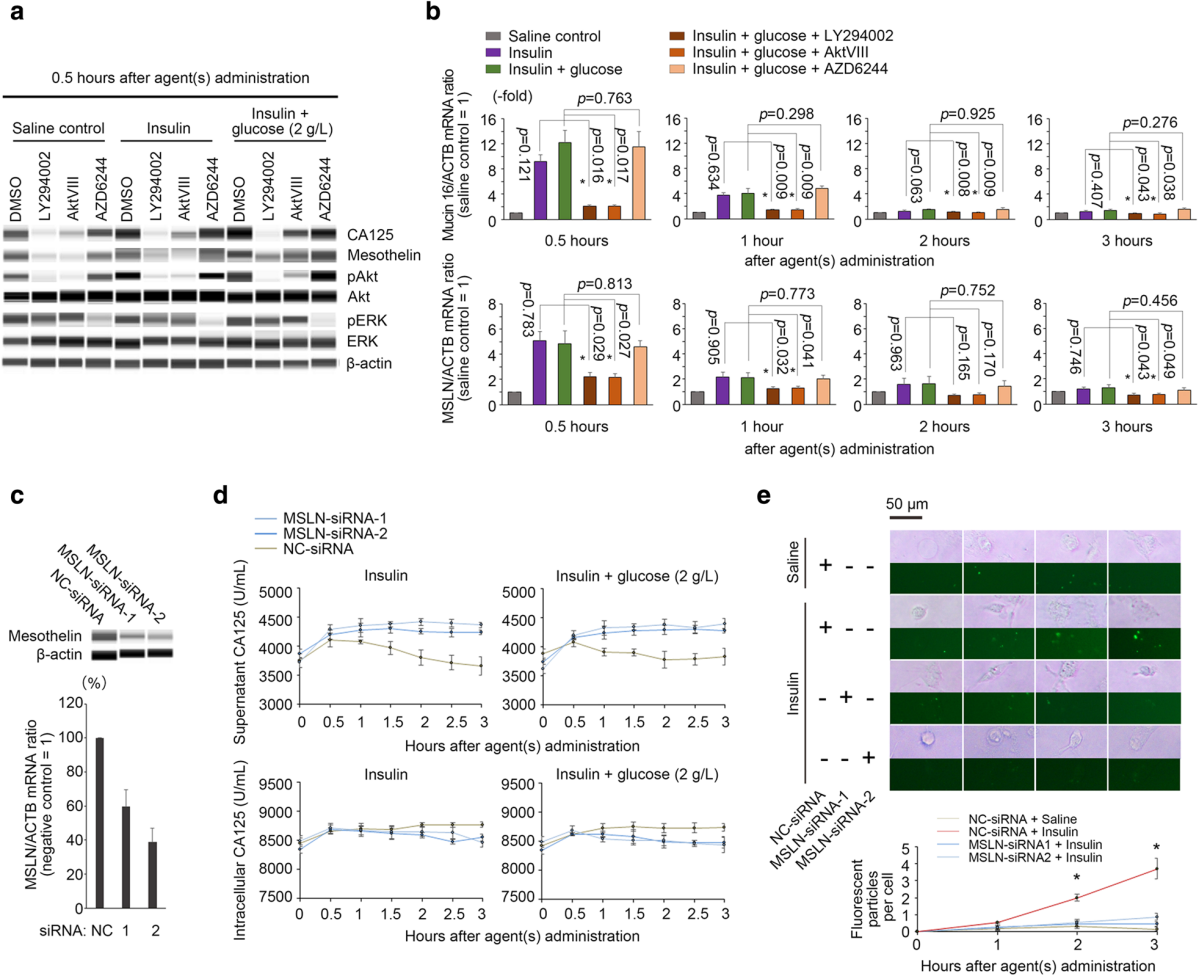
Roles of intracellular signaling pathways and Mesothelin in glucose and insulin-invoked CA125 metabolism. **a** The computational simulation of Western blotting performed using the automatic machine “Wes”. Shown are the intracellular CA125 and Mesothelin protein levels after administration of saline, insulin and glucose plus insulin in ovarian cancer cells (OVCAR-3) with or without PI3K-Akt and MEK-ERK pathway blockades (for details, see Additional file 2: Figure S2). **b** The relative intracellular Mucin16 and MSLN mRNA levels after administration of saline, insulin and glucose plus insulin with or without PI3K-Akt and MEK-ERK blockades (note: Mucin 16/ACTB and MSLN/ACTB mRNA ratios were set to 1 for the saline controls). **c** Knockdown effects of MSLN-specific or NC siRNAs on target protein expression. **d** The time-dependent extracellular and intracellular CA125 levels after MSLN knockdown. **e** The time-dependent re-absorption of Alexa Fluor 488-labeled CA125 after MSLN knockdown. Only the cells treated with NC-siRNA and supplemented with insulin showed significantly increased re-absorption of CA125 protein. *Statistical significance (two-sided Student’s t test)

To further elucidate the molecular basis of glucose and insulin-induced CA125 synthesis/secretion, we investigated the intracellular signaling pathways that could be responsible. Western blotting and RT-qPCR experiments showed that the addition of insulin activated the expression of CA125 and its surface receptor, Mesothelin (encoded by MSLN), at both protein and mRNA levels (Fig. 5a, b). Moreover, the administration of PI3K-Akt inhibitors, LY294002 and AktVIII, but not an MEK-Erk inhibitor, AZD6244, substantially blocked the inductive effect of insulin on CA125 and Mesothelin expression, demonstrating PI3K-Akt pathway as a major transducer between insulin signals and target gene transcription (Fig. 5a, b). Co-administration of glucose and insulin enhanced the activity of the PI3K-Akt pathway and, subsequently, the expression of CA125 and Mesothelin (Fig. 5a, b). However, this enhancing effect did not reach statistical significance at the transcription level (Fig. 5b and Additional file 2: Figure S2), implying that the CA125 concentration peak in the supernatant was most likely regulated via glucose metabolism-related mechanisms at the protein level. We next examined whether elevated Mesothelin (MSLN) expression is a major cause of insulin-induced late-stage decrease in the supernatant CA125 as well as the paradoxical accumulation of CA125 protein in the cytoplasm (Fig. 4a). Two MSLN-specific siRNAs were constructed and introduced into ovarian cancer cells (Fig. 5c). As Mesothelin expression was knocked down, the monotonically ascending curve of intracellular CA125 levels was replaced by a single peak curve (Fig. 5d), reflecting the transience of insulin-induced CA125 expression. Correspondingly, CA125 levels in the supernatant did not show a descending trend; instead, they presented as a sustained ascending curve (Fig. 5d). When Alexa Fluor 488-labeled CA125 protein was added, there were significantly fewer fluorescent particles observed in the MSLN-knockdown cells (Fig. 5e). Co-administration of glucose and insulin to the MSLN-knockdown cells corresponded to a similar ascending curve for the CA125 supernatant level and a single peak curve for intracellular CA125 levels (Fig. 5d). Additionally, the CA125 re-absorption activity of these cells decreased under the high glucose and insulin condition (Additional file 2: Figure S3).

## Discussion

In this study, the enrolled population was a typical representation of patients encountered in a gynecological clinical condition. The gynecological diseases they suffered included four major classes of benign cysts, i.e., pelvic inflammatory cysts, adnexal retention cysts, ovarian endometrioma and benign/borderline cystadenoma, which need to be discriminated from early-stage ovarian cancer due to the high-risk manifestations, such as long-term abdominal pain (inflammatory cysts), progressive CA125 elevation (ovarian endometrioma, benign/borderline cystadenoma), rapid tumor enlargement (retention cysts) and postmenopausal pelvic mass (retention cysts) [6, 7, 23, 24]. Among these diseases, for the first time, we demonstrated that ovarian cancer possesses a unique, distinctive pattern of postprandial increases in serum CA125. By using this increase as a surrogate biomarker, with or without the SVM-modified algorithm, early-stage ovarian cancer detection can be significantly improved (Table 1).

Accepted as a basic carcinogenesis theory, earlier studies have indicated that the PI3K-Akt pathway is frequently altered in cancer cells, which exhibits far stronger activity than it does in healthy cells [25, 26]. For instance, in ovarian cancer, a gain-of-function PI3KCA single nucleotide alteration (SNA) and a PTEN copy number deletion are common gene mutations that cause enriched phosphatidylinositol 3,4,5-trisphosphate (PIP3) and intensified pAkt signaling [26, 27]. Likewise, in our study, the data demonstrated that it is the PI3K-Akt pathway that invokes CA125 expression (Fig. 5a, b) and distinguishes the postprandial pattern of cancer-derived CA125 from those observed in benign diseases (Figs. 1 and 2). Mechanistically, the serum CA125 test works in a static mode, which provides rather limited information on the source of CA125. The only way to increase the specificity of this test is to increase the cut-off, e.g., 65 U/mL [5]. However, for malignant cysts< 5 cm, this strategy is often less effective (Fig. 1a, b). In contrast, the CA125 postprandial increment test provides a dynamic strategy for discerning subtle differences of PI3K-Akt signaling pathway among various types of ovarian (adnexal) cysts. Therefore, the properties of unidentified cysts can be determined at a lower CA125 cut-off level, i.e., 20 U/mL, decreasing the missed diagnosis rate imposed by the original cut-off of 35 U/mL (Table 1).

Our animal and cancer cell models both revealed that, insulin, only when combined with a high-glucose condition can invoke the active release of CA125; otherwise, it promotes re-absorption of CA125 (Figs. 3 and 4). The high-glucose condition, hence, plays a critical role in switching the function of insulin. Theoretically, insulin facilitates the recycling of GLUT4, a key glucose transporter, in ovarian cancer [28]; in turn, the accumulated intracellular glucose, through glycolysis and the Kreb’s cycle, offers an abundant reservoir of glucose-6-phosphate and energy molecules to the glycosyltransferase system that is responsible for CA125-core glycosylation [29, 30]. The accelerated CA125 synthesis/secretion at the protein level, therefore, is attainable only under a high-glucose condition. This fact was manifested by the post-75-g oral glucose tests in the enrolled population, which revealed an ascending region of serum CA125 coincidentally accompanying the elevated insulin level and the consumption of blood glucose resources (Fig. 1d). Mesothelin, however, as another factor affected by insulin, contributes in the opposite manner (Fig. 5d). Previous studies demonstrated that a blockade of the interaction between Mesothelin and CA125 (using an MORAb-009 antibody) led to a 1.3- to 13.75-fold increase in CA125 in patient sera [31]. We, herein, demonstrated that overexpressed Mesothelin mediates 1.1- to 19.4-fold greater CA125 re-absorption in insulin-treated cancer cells compared with that in control cells (Fig. 5e), while Mesothelin overexpression itself is also modulated by the PI3K-Akt pathway (Fig. 5a, b). Moreover, there was a characteristic over-drop segment of postprandial CA125 observed in the non-cancer patients (Fig. 1d); and in patients with a pelvic inflammatory cyst, an inverse relationship between average fasting and postprandial CA125 was established (Fig. 1b). With the above Mesothelin-based mechanism, we can reasonably attribute these phenomena to the vast Mesothelin-expressing system of peritoneal mesothelium, where CA125 re-absorption events can actively occur, and the glucose and insulin-invoked responses for CA125 synthesis/secretion may be insufficient [32]. Additionally, considering that a number of monocytes and resident macrophages may infiltrate the inflammatory cyst tissue [33], Siglec family proteins (i.e., Siglec-2, 7 and 9), which are an additional panel of monocyte/macrophage-specific CA125 receptors [34–36], may also skew the CA125 secretion/re-absorption balance toward a decrease in extracellular CA125. As supporting evidence, we have confirmed an effect of glucose and insulin on the induction of Siglec-9 overexpression in the peritoneal resident macrophages (data not shown).

Although in this work, by including the postprandial increase in the serum CA125 test, the CA125 cut-off was successfully lowered and the efficacy of early-stage ovarian cancer detection was accordingly improved, we still noted that there were cancer cases missed, even after adopting a postprandial increment-based diagnostic criterion with the highest efficacy (i.e., CA125 increment ≥ 10%). Most of these cases (5/6) were characterized by CA125 > 105 U/mL (Fig. 2e), which means that the CA125 increment ≥ 10% rule is not applicable to the entire CA125 level space, especially for higher CA125 values. In other words, the relationship between fasting and postprandial CA125 levels should have abided by a non-linear rule in early-stage ovarian cancer. These conditions necessitated the adoption of an SVM in our study. The kernel function is the foundation of the SVM, and we selected the RBF, which requires fewer input parameters and exhibits higher stability [20]. Our results demonstrated the utility of the SVM in improving diagnostic quality of postprandial increases in CA125 (Table 1 and Fig. 2f) and an ideal usage of these two detection tools in the clinical practice.

A limitation of this work is that the food-intake of each patient was randomized, which has not yet been standardized. Certainly, a quantitative glucose uptake test, such as an OGTT and an intravenous glucose tolerance test (IVGTT), would be beneficial to gain a more stable, reliable, and informative dataset on the postprandial CA125 fluctuation patterns in cancer and non-cancer patients. Therefore, further efforts should focus on investigating the applicability of standard OGTT (or IVGTT)-associated fluctuation pattern(s) of cancer-derived serum CA125 in large-scale patient populations.

## Conclusions

In conclusion, the present study adopted the concepts of “glucose and insulin-induced CA125” and “dynamic measurement/assessment of serum cancer biomarkers”. Moreover, by exploiting the subtle differences in postprandial increases in serum CA125 among patients with endometriosis and benign and malignant ovarian tumors, an SVM-based CA125-increment algorithm was built. This algorithm exhibited improved efficacy on detecting early-stage ovarian cancer. Additionally, to a certain degree, this work provides a paradigm for future explorations of the dynamic and inductive behaviors of more serum cancer biomarkers, such as HE4, CA199, CEA, and PSA.

## Additional files

**Additional file 1: Table S1–S5.** Demographic, clinical and pathological characteristics of the study population. Serum CA125 levels (random) detected in the study population. Comparisons of the differences of CA125 serum levels (random) among the five disease groups. Comparisons of the differences between fasting and postprandial CA125 serum levels for the five disease groups. Coordinates of the ROC curve for fasting CA125 levels (calculated by SPSS 18.0).

**Additional file 2: Figure S1–S3.** Comparisons of the fluctuation patterns of blood glucose, serum insulin and CA125 between the ovarian cancer and non-cancer patients. Comparisons of relative CA125 and Mesothelin protein levels between ovarian cancer cells (OVCAR-3) treated with different agents, namely DMSO, insulin, insulin with high glucose, and insulin with high glucose and PI3K-Akt/MEK-Erk inhibitors. The time-dependent re-absorption of Alexa Fluor 488-labeled CA125 by ovarian cancer cells (OVCAR-3) under the high-glucose plus insulin condition.

## Abbreviations

ACTB: β-actin
AUC: area under the receiver operating characteristic curve
CEA: carcino-embryonic antigen
CT: cycle threshold
EGF: epithelial growth factor
HE4: human epididymis protein 4
i.h.: injectio hypodermatica
IVGTT: intravenous glucose tolerance test
MSLN: mesothelin
NC: negative control
NPV: negative predictive value
OGTT: oral glucose tolerance test
PIP3: phosphatidylinositol 3,4,5-trisphosphate
p.o.: per os
PPV: positive predictive value
PSA: prostate specific antigen
PTEN: phosphatase and tensin homolog
qPCR: quantitative polymerase chain reaction
QUADA-2: quality assessment of diagnostic accuracy studies-2
RBF: radial basis function
ROC: receiver operating characteristic
RT: reverse transcription
SNA: single nucleotide alteration
SPF: specific pathogen-free
SVM: support vector machine
TVU: transvaginal ultrasonography
