# Supplementary material for: Postprandial increase in serum CA125 as a surrogate biomarker for early diagnosis of ovarian cancer
Source: J Transl Med. 2018 May 1;16:114. doi: 10.1186/s12967-018-1489-4 (PMC5930842; doi:10.1186/s12967-018-1489-4)
Supplement: Supplementary file 1 — Additional file 1: Table S1–S5. Demographic, clinical and pathological characteristics of the study population. Serum CA125 levels (random) detected in the study population. Comparisons of the differences of CA125 serum levels (random) among the five disease groups. Comparisons of the differences between fasting and postprandial CA125 serum levels for the five disease groups. Coordinates of the ROC curve for fasting CA125 levels (calculated by SPSS 18.0). [file 12967_2018_1489_MOESM1_ESM.pdf]

Additional File 1

Table S1. Demographic, clinical and pathological characteristics of the study population.

Characteristics	Pelvic inflammatory cyst (n=55)	Adnexal retention cyst (n=65)	Ovarian endometrioma (n=348)	Ovarian benign/borderline cystadenoma (n=47)	Ovarian cancer (n=36)	p value
Age (years)*	36.1 ± 6.9	52.6 ± 12.8	36.9 ± 6.9	40.7 ± 11.5	54.3 ± 6.9	< 0.001
Gravity*	1.1 ± 1.2	2.2 ± 1.1	1.4 ± 1.0	1.7 ± 1.1	2.0 ± 0.9	< 0.001
Parity*	0.4 ± 0.6	1.2 ± 0.6	0.7 ± 0.6	0.8 ± 0.5	1.0 ±0.4	< 0.001
Cyst size (mm)**	4.3 ± 0.5	4.4 ± 0.4	4.2 ± 0.5	4.3 ± 0.4	4.3 ± 0.5	0.065
Ascites						-
Yes	-	-	-	0	0	
No	-	-	-	47	36	
Peritoneal metastasis						-
Yes	-	-	-	0	0	
No	-	-	-	47	36	
Lymphatic metastasis						-
Yes	-	-	-	0	0	
No	-	-	-	47	36	
Stage†						-
I	-	-	-	1	23	
II	-	-	-	0	13	
III	-	-	-	0	0	
IV	-	-	-	0	0	
Histotypes‡						0.032
Serous	-	-	-	44	29	
Mucinous	-	-	-	3	1	
Endometrioid	-	-	-	0	4	

Clear cell	-	-	-	0	2	
Grade						-
G1	-	-	-	-	9	
G2	-	-	-	-	12	
G3	-	-	-	-	15	

* Data are presented as the mean ± standard deviation, ANOVA.

** Measured as the maximal diameter of the cyst.

† FIGO 2000 (for borderline cystadenoma and ovarian cancer only).

‡ Only patients with ovarian benign/borderline cystadenoma and patients with ovarian cancer were compared, two-sided χ^2 test.

Table S2. Serum CA125 levels (random) detected in the study population.

Disease types	Patient number	Median (U/mL)	Mean (U/mL)	Standard deviation (U/mL)
Pelvic inflammatory cyst	55	35.5	109.2	170.2
Adnexal retention cyst	65	15.2	23.3	26.0
Simple cyst	38	14.3	21.7	18.6
Mesonephric cyst	27	15.2	25.4	34.1
Endometriotic cyst	348	87.5	103.1	60.1
Benign/borderline cyst	47	105.5	120.1	91.6
Serous cystadenoma	42	102.3	115.9	91.6
Mucinous cystadenoma	3	81.5	104.8	88.8
Borderline serous cystadenoma	2	231.3	231.3	25.1
Ovarian cancer	36	94.6	127.1	113.4
Serous	29	88.1	124.1	118.2
Stage I	19	88.1	123.2	109.2
Stage II	10	87.3	125.8	139.9
Mucinous	1	312.5	312.5	-
Stage II	1	312.5	312.5	-
Endometrioid	4	72.6	102.0	82.8
Stage I	3	56.0	61.8	24.8
Stage II	1	222.5	222.5	-
Clear cell	2	128.1	128.1	32.5
Stage I	1	105.1	105.1	-
Stage II	1	151.1	151.1	-
Stage I	23	88.5	114.5	101.3
Stage II	13	110.6	149.5	133.5

Table S3. Comparisons of the differences in CA125 serum levels (random) among the five disease groups.*

	Pelvic inflammatory cyst	Adnexal retention cyst	Ovarian endometrioma	Ovarian benign/borderline cystadenoma	Ovarian cancer
Pelvic inflammatory cyst	-	<0.001	0.788	0.699	0.582
Adnexal retention cyst	-	-	<0.001	<0.001	<0.001
Ovarian endometrioma	-	-	-	0.222	0.217
Ovarian benign/borderline cystadenoma	-	-	-	-	0.754
Ovarian cancer	-	-	-	-	-

* For each pair of disease groups, a p value for the difference of their CA125 serum levels (random) was given. Two-sided Student's t test (for independent samples) was used.

Table S4. Comparisons of the differences between fasting and postprandial CA125 serum levels for the five disease groups.

Groups	Fasting CA125 (U/mL)†	Postprandial CA125 (U/mL)†	CA125 increment (%)†‡	*p* value
Pelvic inflammatory cyst	110.9 ± 181.1	107.3 ± 159.8	1.3 ± 7.0	0.322
Adnexal retention cyst	22.8 ± 25.2	23.6 ± 26.8	2.7 ± 8.1	0.007
Ovarian endometrioma	100.9 ±58.8	104.3 ± 60.9	3.2 ± 6.4	< 0.001
Ovarian benign/borderline cystadenoma	119.7 ± 93.5	120.4 ± 90.7	3.4 ± 9.3	0.808
Ovarian cancer	118.4 ± 108.9	131.6 ± 118.0	13.3 ± 6.9	< 0.001

† Data are presented as the mean ± standard deviation, paired-samples Student's t test (two-sided).

‡ For each patient, the CA125 increment = (fasting CA125 – postprandial CA125) / fasting CA125 × 100%.

Table S5. Coordinates of the ROC curve for fasting CA125 levels (calculated by SPSS 18.0).

Positive if greater than or equal to the CA125 level (U/mL)	Sensitivity	1 - Specificity
3.130	1.000	1.000
4.765	1.000	.998
5.570	1.000	.996
6.420	1.000	.994
7.200	1.000	.992
7.350	1.000	.990
7.420	1.000	.988
7.470	1.000	.986
7.750	1.000	.984
8.065	.972	.983
8.215	.972	.981
8.330	.972	.977
8.825	.972	.975
9.295	.972	.973
9.350	.972	.969
9.450	.972	.967
9.850	.972	.965
10.250	.972	.963
10.350	.972	.959
10.600	.972	.957
10.950	.972	.955
11.200	.972	.953
11.350	.972	.946
11.450	.972	.944
11.950	.972	.942
12.450	.972	.938
12.650	.972	.936
12.800	.972	.934
12.890	.972	.930
12.990	.972	.928
13.030	.944	.926
13.270	.944	.924
13.840	.944	.922
14.220	.944	.915
14.320	.944	.913
14.750	.944	.911
15.250	.944	.909
15.450	.944	.905
15.535	.944	.903
15.935	.944	.901
16.400	.944	.899
16.550	.944	.897

16.650	.944	.895
16.900	.944	.893
17.150	.944	.891
17.250	.944	.889
17.405	.944	.887
17.555	.944	.885
17.800	.944	.883
18.005	.917	.883
18.055	.917	.882
18.150	.917	.880
18.550	.917	.878
19.000	.917	.876
19.200	.917	.874
19.590	.917	.872
19.940	.917	.870
20.050	.889	.870
20.250	.889	.866
20.750	.889	.864
21.105	.889	.862
21.155	.889	.860
21.250	.889	.858
21.350	.889	.856
21.450	.889	.854
21.900	.889	.847
22.350	.889	.845
22.450	.889	.843
22.800	.889	.841
23.200	.889	.837
23.350	.889	.835
23.750	.889	.833
24.200	.889	.831
24.700	.889	.827
25.200	.889	.825
25.350	.889	.821
25.490	.889	.819
25.590	.889	.817
25.650	.889	.816
25.740	.889	.814
25.890	.861	.814
26.050	.833	.814
26.200	.833	.812
26.330	.833	.808
26.380	.833	.806
26.450	.833	.804
26.800	.833	.802
27.100	.806	.802
27.150	.806	.800

27.250	.806	.798
27.350	.806	.796
27.550	.778	.794
27.850	.778	.792
28.050	.778	.790
28.150	.778	.786
28.350	.778	.784
28.800	.778	.783
29.150	.778	.777
29.250	.778	.775
29.305	.778	.773
29.405	.778	.771
29.955	.778	.769
30.455	.778	.767
30.850	.778	.765
31.485	.778	.763
31.935	.778	.761
32.150	.778	.759
32.250	.778	.757
32.400	.778	.755
32.550	.750	.753
32.900	.750	.751
33.700	.750	.750
34.250	.722	.748
34.400	.722	.744
34.800	.722	.742
35.150	.694	.740
35.250	.694	.734
35.350	.694	.732
35.850	.694	.728
36.500	.694	.724
37.100	.694	.722
37.850	.694	.720
38.250	.694	.709
38.350	.694	.707
38.450	.694	.705
38.685	.694	.703
39.035	.694	.701
39.300	.694	.697
39.450	.694	.695
40.850	.694	.693
42.250	.694	.691
42.700	.667	.689
43.200	.667	.687
43.350	.667	.685
44.300	.667	.683
45.650	.639	.682

46.150	.639	.680
46.250	.639	.678
46.450	.639	.676
46.650	.639	.674
46.950	.639	.672
47.250	.639	.670
47.700	.639	.668
48.600	.639	.666
49.200	.639	.664
49.800	.639	.662
50.700	.639	.660
51.580	.639	.658
52.080	.639	.656
52.200	.639	.654
52.650	.639	.650
53.150	.639	.649
53.350	.639	.647
53.450	.639	.643
53.800	.639	.641
54.150	.639	.639
54.350	.639	.637
55.400	.639	.635
56.400	.639	.633
56.600	.639	.631
56.850	.639	.629
57.100	.639	.627
57.300	.639	.625
57.500	.639	.623
57.850	.639	.619
58.550	.639	.617
59.050	.639	.616
59.150	.639	.610
59.300	.639	.608
59.450	.611	.608
60.450	.611	.606
61.700	.611	.604
62.050	.611	.602
62.200	.611	.600
62.700	.611	.598
63.200	.611	.596
63.350	.611	.592
63.450	.611	.590
64.050	.611	.588
64.900	.611	.586
65.250	.611	.584
65.750	.611	.583
66.300	.611	.577

66.750	.611	.575
67.150	.611	.573
67.250	.583	.567
67.350	.583	.563
67.450	.583	.559
67.800	.583	.557
68.250	.556	.557
68.550	.556	.553
68.850	.556	.551
69.100	.556	.550
69.300	.556	.548
69.800	.556	.544
70.350	.556	.540
70.900	.556	.538
71.700	.556	.536
72.200	.556	.534
72.450	.556	.530
72.850	.556	.528
73.150	.556	.526
73.250	.556	.522
73.450	.556	.520
74.000	.556	.518
74.450	.556	.517
74.750	.556	.515
75.050	.556	.513
75.200	.556	.511
75.350	.556	.507
75.450	.556	.503
76.000	.556	.501
76.850	.556	.497
77.250	.528	.497
77.750	.528	.495
78.250	.500	.495
78.350	.500	.491
78.750	.500	.485
79.200	.500	.480
79.350	.500	.478
79.450	.500	.476
79.800	.500	.474
80.250	.500	.472
80.700	.500	.470
81.250	.500	.468
81.750	.500	.466
82.100	.500	.464
82.250	.500	.462
82.350	.500	.458
82.450	.500	.456

82.600	.500	.454
82.900	.500	.452
83.650	.500	.450
84.250	.500	.449
84.350	.500	.445
84.750	.500	.443
85.150	.500	.441
85.700	.500	.439
86.650	.500	.437
87.150	.500	.433
87.250	.500	.431
87.350	.500	.429
87.950	.500	.427
88.750	.472	.427
89.050	.472	.425
89.150	.472	.423
89.350	.472	.421
89.550	.472	.417
89.800	.472	.416
90.100	.472	.412
90.300	.472	.410
90.500	.472	.406
90.850	.472	.404
91.150	.472	.402
91.350	.472	.400
91.800	.472	.398
92.150	.472	.396
92.250	.472	.394
92.700	.472	.390
93.150	.472	.388
93.600	.472	.384
94.100	.472	.383
94.300	.472	.381
94.850	.472	.379
95.350	.472	.377
95.800	.472	.375
96.350	.472	.373
96.550	.472	.371
96.950	.472	.369
97.500	.444	.363
98.000	.417	.363
98.350	.417	.361
98.450	.417	.357
98.750	.417	.355
99.100	.417	.353
99.300	.417	.348
100.350	.417	.346

101.750	.417	.344
102.350	.417	.340
102.900	.417	.338
104.250	.417	.336
105.250	.389	.336
105.450	.389	.334
105.700	.389	.332
106.550	.389	.330
107.350	.389	.328
107.500	.361	.328
107.850	.361	.326
108.250	.361	.324
108.750	.361	.322
109.250	.361	.317
109.950	.361	.315
110.800	.361	.313
111.600	.361	.311
112.200	.361	.309
112.350	.361	.307
113.450	.361	.305
114.800	.361	.303
115.250	.361	.301
116.350	.361	.299
117.350	.361	.297
117.450	.361	.295
117.600	.361	.293
119.255	.361	.291
121.055	.361	.289
121.350	.361	.283
121.800	.361	.282
122.300	.361	.280
122.450	.361	.278
122.950	.361	.276
123.850	.361	.274
124.450	.361	.272
124.950	.361	.270
125.350	.361	.268
125.450	.361	.266
125.590	.361	.264
125.690	.361	.262
125.900	.361	.260
126.150	.361	.258
126.800	.361	.256
127.900	.361	.252
128.450	.333	.252
128.750	.333	.250
129.050	.333	.249

129.150	.333	.245
129.850	.333	.243
130.950	.306	.243
131.950	.306	.241
132.900	.306	.239
134.400	.306	.237
135.950	.306	.235
137.400	.306	.233
138.700	.306	.231
139.050	.278	.231
139.150	.278	.229
139.300	.278	.227
140.300	.278	.225
141.800	.278	.223
142.500	.278	.219
143.150	.278	.217
144.950	.278	.216
147.250	.250	.216
148.650	.222	.216
149.100	.222	.214
149.750	.222	.210
151.150	.222	.208
152.100	.222	.206
152.350	.222	.204
152.900	.222	.202
153.700	.222	.200
154.250	.222	.198
154.700	.222	.196
155.150	.222	.194
155.650	.222	.192
156.050	.222	.190
156.150	.222	.188
157.800	.222	.186
159.800	.222	.184
160.650	.222	.183
161.800	.222	.181
162.900	.222	.179
163.750	.222	.177
165.700	.222	.173
167.850	.222	.171
169.350	.222	.169
170.700	.222	.167
172.200	.222	.165
174.350	.222	.163
175.550	.222	.161
176.500	.222	.159
177.700	.222	.157

178.350	.222	.155
178.900	.222	.153
179.200	.222	.151
179.750	.222	.148
180.300	.222	.146
180.850	.222	.144
181.750	.222	.142
182.250	.222	.140
182.650	.222	.138
183.050	.222	.136
183.150	.222	.134
183.750	.222	.132
184.350	.222	.130
184.450	.222	.128
184.750	.222	.126
185.650	.222	.124
186.650	.222	.122
187.100	.222	.120
188.150	.222	.118
189.250	.222	.117
191.300	.222	.115
193.250	.222	.113
193.400	.222	.111
194.950	.222	.109
197.000	.222	.107
197.950	.222	.105
198.650	.222	.103
199.050	.222	.101
200.150	.222	.099
201.600	.222	.097
202.500	.222	.095
203.550	.222	.093
204.350	.222	.091
205.950	.222	.089
207.700	.222	.087
208.250	.222	.085
208.450	.222	.083
208.850	.194	.083
209.250	.194	.082
209.400	.194	.080
211.400	.194	.078
213.650	.194	.076
214.200	.194	.074
214.750	.194	.072
215.300	.194	.070
216.450	.194	.068
219.350	.194	.066

221.350	.194	.064
223.200	.194	.062
225.600	.194	.060
226.750	.194	.058
227.400	.194	.056
229.250	.194	.054
231.100	.194	.052
231.250	.194	.050
231.350	.194	.049
232.300	.194	.047
234.300	.194	.045
236.350	.194	.043
239.200	.194	.041
241.700	.194	.039
242.750	.194	.037
245.450	.194	.035
248.450	.194	.033
251.400	.194	.031
253.900	.167	.031
256.750	.167	.029
259.800	.167	.027
260.850	.167	.025
262.900	.167	.023
265.700	.139	.023
267.150	.139	.021
268.800	.111	.021
270.400	.111	.019
272.500	.111	.017
283.400	.111	.016
294.250	.111	.014
298.700	.083	.014
301.750	.056	.014
302.350	.056	.012
307.550	.056	.010
317.000	.056	.008
330.250	.056	.006
340.800	.056	.004
353.400	.056	.002
394.800	.028	.002
818.200	.000	.002
1212.200	.000	.000
