# Supplementary material for: Postprandial increase in serum CA125 as a surrogate biomarker for early diagnosis of ovarian cancer
Source: J Transl Med. 2018 May 1;16:114. doi: 10.1186/s12967-018-1489-4 (PMC5930842; doi:10.1186/s12967-018-1489-4)
Supplement: Supplementary file 2 — Additional file 2: Figure S1–S3. Comparisons of the fluctuation patterns of blood glucose, serum insulin and CA125 between the ovarian cancer and non-cancer patients. Comparisons of relative CA125 and Mesothelin protein levels between ovarian cancer cells (OVCAR-3) treated with different agents, namely DMSO, insulin, insulin with high glucose, and insulin with high glucose and PI3K-Akt/MEK-Erk inhibitors. The time-dependent re-absorption of Alexa Fluor 488-labeled CA125 by ovarian cancer cells (OVCAR-3) under the high-glucose plus insulin condition. [file 12967_2018_1489_MOESM2_ESM.pdf]

Additional File 2

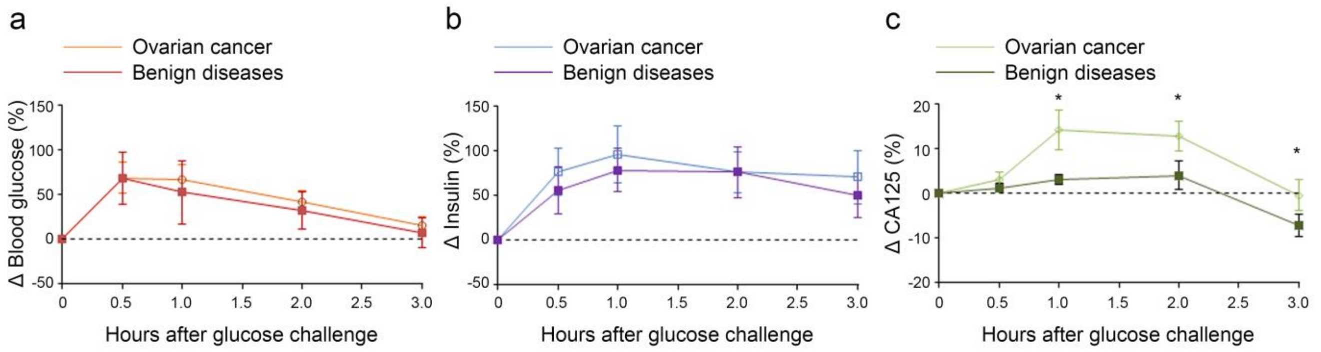

Figure S1. Comparisons of the fluctuation patterns of blood glucose, serum insulin and CA125 between the ovarian cancer and non-cancer patients. **a** The Δblood glucose curves. **b** The Δinsulin curves. **c** The ΔCA125 curves. Patients with benign diseases or ovarian cancer had similar glucose and insulin fluctuation patterns, while their CA125 fluctuation patterns were distinctly different. Besides, a segment that significantly dropped (over-drop) below the fasting CA125 level (i.e., the reference line) was noted in the ΔCA125 curve of the benign disease group. *, statistical significance (two-sided Student's t test).

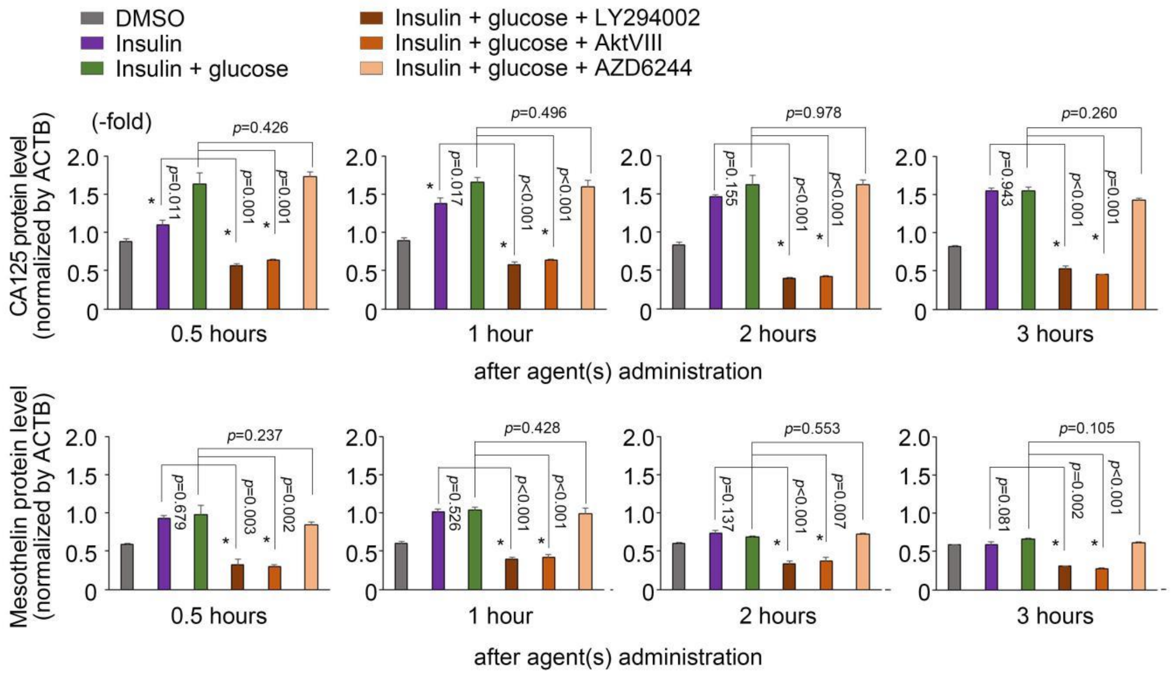

Figure S2. Comparisons of relative CA125 and Mesothelin protein levels between ovarian cancer cells (OVCAR-3) treated with different agents, namely DMSO, insulin, insulin with high glucose, and insulin with high glucose and PI3K-Akt/MEK-Erk inhibitors. The protein expression data of CA125 and Mesothelin were calculated based on the digitalized Western blotting results derived using the "Wes" machine. Shown are the β-actin (ACTB)-normalized CA125 and Mesothelin protein levels at the post-treatment timepoints of 0.5, 1, 2 and 3 hours. The expression level of the high-glucose and insulin-invoked CA125 was significantly higher than that of the insulin only-induced CA125 at 0.5 hours after treatment. The induced CA125 expression was suppressed by PI3K-Akt inhibitors (Ly294002 and AktVIII) but not the MEK-Erk inhibitor AZD6244. The additional increase in CA125 expression observed in the high-glucose condition was sustained for only 1 hour in the treated cancer cells. From the 2nd hour through the 3rd hour after treatment, the CA125 protein levels in the cells of the insulin-only administration group and the insulin plus glucose administration group become tantamount to each other. *, statistical significance (two-sided Student's t test).

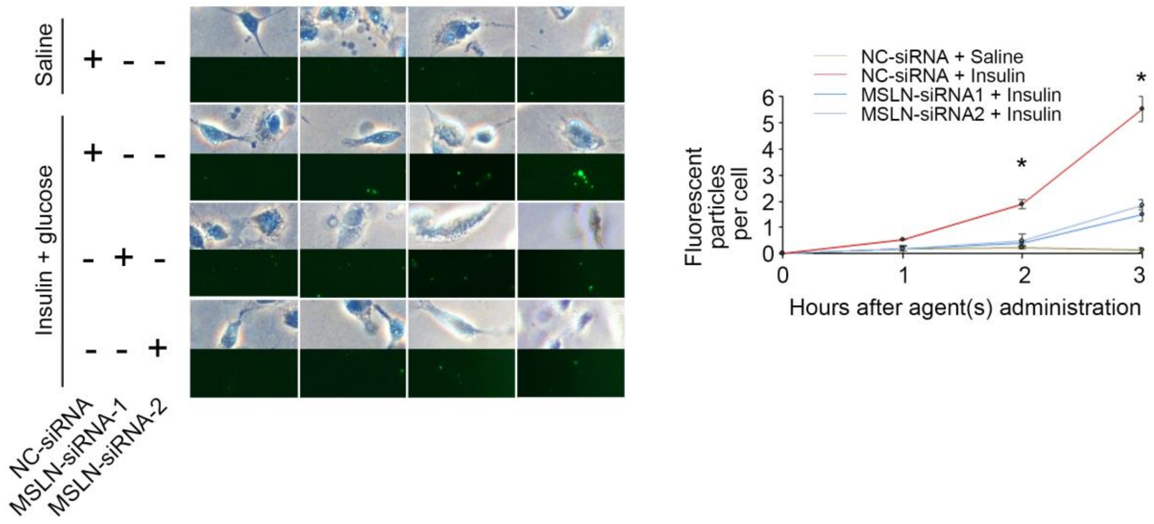

Figure S3. The time-dependent re-absorption of Alexa Fluor 488-labeled CA125 by ovarian cancer cells (OVCAR-3) under the high-glucose plus insulin condition. Shown are the cells transfected with NC-siRNA, MSLN-siRNA-1 and MSLN-siRNA-2. Cell cultures administered saline were treated with the transfection agent at the same dose as that used for negative control and MSLN-specific siRNAs. The fluorescent particles were counted at 200×magnification under a microscope, and the per cell particle number was presented as the mean ± SD based on the counts of 60 cells from three replicates (i.e., 20 cells each). *, statistical significance (two-sided Student's t test).
